# Metabolite profile of COVID-19 revealed by UPLC-MS/MS-based widely targeted metabolomics

**DOI:** 10.3389/fimmu.2022.894170

**Published:** 2022-07-18

**Authors:** Jun Liu, Zhi-Bin Li, Qi-Qi Lu, Yi Yu, Shan-Qiang Zhang, Pei-Feng Ke, Fan Zhang, Ji-Cheng Li

**Affiliations:** ^1^ Medical Research Center, Yue Bei People’s Hospital, Shantou University Medical College, Shaoguan, China; ^2^ The Central Laboratory, Yangjiang People’s Hospital, Yangjiang, China; ^3^ Department of Histology and Embryology, Shaoguan University School of Medicine, Shaoguan, China; ^4^ Institute of Cell Biology, Zhejiang University School of Medicine, Hangzhou, China

**Keywords:** COVID-19, potential biomarkers, widely targeted metabolites, machine learning, UPLC-MS/MS

## Abstract

The metabolic characteristics of COVID-19 disease are still largely unknown. Here, 44 patients with COVID-19 (31 mild COVID-19 patients and 13 severe COVID-19 patients), 42 healthy controls (HC), and 42 patients with community-acquired pneumonia (CAP), were involved in the study to assess their serum metabolomic profiles. We used widely targeted metabolomics based on an ultra-performance liquid chromatography–tandem mass spectrometry (UPLC-MS/MS). The differentially expressed metabolites in the plasma of mild and severe COVID-19 patients, CAP patients, and HC subjects were screened, and the main metabolic pathways involved were analyzed. Multiple mature machine learning algorithms confirmed that the metabolites performed excellently in discriminating COVID-19 groups from CAP and HC subjects, with an area under the curve (AUC) of 1. The specific dysregulation of AMP, dGMP, *sn*-glycero-3-phosphocholine, and carnitine was observed in the severe COVID-19 group. Moreover, random forest analysis suggested that these metabolites could discriminate between severe COVID-19 patients and mild COVID-19 patients, with an AUC of 0.921. This study may broaden our understanding of pathophysiological mechanisms of COVID-19 and may offer an experimental basis for developing novel treatment strategies against it.

## Introduction

The COVID-19 pandemic remains an ongoing critical threat. As of January 20, 2022, 336,790,193 confirmed cases worldwide, including 5,560,718 deaths, have been reported by the World Health Organization (https://covid19.who.int/). Although COVID-19 has been effectively controlled in China, sporadic cases have emerged that counteracted the controlling efforts. In fact, in a 2021 *Nature* poll, many prominent experts expressed their opinion on the future of the SARS-CoV-2 virus, and nearly 90% believed that it would continue spreading globally for years to come (1).

Viral infections perturb the host metabolism, and the outcome depends on the stage of viral infection (2). Using mass spectrometry-based metabolomics, Shen et al. identified that the levels of over 100 lipid metabolites were abnormal during COVID-19 infection (3). The perturbed metabolites were mainly related to activation of the complement system, macrophage function, and platelet degranulation. Tiffany Thomas et al. reported that COVID-19 infection could trigger changes in host tryptophan metabolic pathways, and this metabolic reprogramming is also related to inflammation and immune regulation *in vivo* (4). However, our knowledge of the changes of host metabolic pathways after COVID-19 infection is still very limited. In addition, the mechanism driving metabolite change during the infection is poorly understood. Metabolomics is a relatively new method that quantifies metabolites in a biological sample and explores the occurrence and development of human diseases (5, 6). Unlike other omics approaches, it is highly conserved and endogenous, having a high potential to discover biomarkers. Most metabolomic studies of COVID-19 were nontargeted, but this method has certain limitations in identifying metabolites. Therefore, more advanced and reliable technology for identifying and quantifying metabolites is paramount to understand the metabolic changes in patients with COVID-19.

In this study, we first employed a widely targeting technology based on Metabolon ultra-performance liquid chromatography–tandem mass spectrometry (UPLC-MS/MS) to determine the global metabolic profile of patients with COVID-19. Next, we explored whether the COVID-19 infection can induce disturbances in metabolite abundance in order to explore its underlying pathophysiology.

## Materials and methods

### Blood sample collection and clinical data

The study was conducted according to the Declaration of Helsinki and was approved by the Ethics Committee of the First Affiliated Hospital of Zhejiang University School of Medicine, China. All patients and healthy volunteers signed the informed consent, and their fasting blood samples were collected in the morning hours. The diagnosis of CAP was based on the physical examination, clinical manifestations, and chest X-ray changes. All patients enrolled in present study were initial diagnosed, and blood sampling were performed before any intervention. A disposable EDTA-anticoagulated vacuum blood collection tube was used to store 5 ml of the freshly drawn peripheral blood. Plasma was separated and stored as aliquots at −80°C and clinical information was recorded (**Table 1**). The samples were further processed in a biosafety level 2 laboratory qualified for SARS-CoV-2 testing following the Laboratory Biosafety Guidelines for COVID-19 (2nd edition) issued by the National Health Commission of China. Plasma samples of 44 patients with COVID-19 (31 mild COVID-19 patients and 13 severe patients), 42 healthy volunteers, and 42 patients with community-acquired pneumonia (CAP) were collected. In addition, clinical information was recorded, and the sample information for all patients and healthy volunteers are presented in **Table 1**.

**Table 1:** The baseline clinical information of both groups.

Characteristic	Mild COVID-19	Severe COVID-19	Healthy controls	Community-acquired pneumonia	*p*-value (Mild vs Severe COVID-19)
n	31	13	42	42	
Sex, n (%)					1.000
Female	15 (48.4%)	6 (46.2%)	20 (47.6%)	20 (47.6%)	
Male	16 (51.6%)	7 (53.8%)	22 (52.4%)	22 (52.4%)	
Age, median (IQR)	41 (35.5, 49)	52 (35, 55)	50 (35, 57)	46 (37.75, 55)	0.086

### Full-spectrum metabolic identification and data acquisition

The plasma metabolites were determined using UPLC-MS/MS (ExionLC AD coupled to a QTRAP spectrometer) (https://sciex.com.cn/). The liquid-phase conditions were as follows: (1) chromatographic separation using Waters Acquity UPLC HSS T3 column (1.8 µm particle size, 100 mm × 2.1 mm; (2) analyte elution on a mobile phase with ultrapure water (0.1% formic acid) for phase A and acetonitrile (0.1% formic acid) for phase B; (3) elution gradients were water/acetonitrile (95:5 V/V) at 0 min, 10:90 V/V at 11.0 min, 10:90 V/V at 12.0 min, 95:5 V/V at 12.1 min, and 95:5 V/V at 14.0 min; (4) the flow rate was 0.4 ml/min, the column temperature was 40°C, and the injection volume was 2 μl. The MS/MS conditions were as follows: 500°C, electrospray ionization; 5500 V (positive) and −4500 V (negative); 55 psi, ion gas source I; 60 psi, gas source II; 25 psi, curtain gas; and high collision-activated dissociation parameters. The ions were scanned and detected according to an optimized decluttering potential and collision energy. They were subjected to qualitative determination based on the retention time, daughter–parent ion pair information, and secondary spectral data of the detected substances using the MetWare database (http://www.metware.cn/). The analytes were quantified using the QTRAP multiple-reaction monitoring mode. After collecting data for different samples, the area under the peak was scored separately for the chromatographic peaks of the extracted ions of all metabolites. Finally, score correction for the chromatographic peaks of the same metabolite in different specimens followed.

### Quality control analysis

Quality control (QC) samples were prepared from the extract mixture of all tested samples and analyzed under the same conditions to monitor the reproducibility of the test. In the process of sample testing, the QC samples were analyzed once every 10 samples to ensure the stability of the analytical process. The repeatability of metabolite extraction and detection can be determined by overlapping analysis for the total ion current (TIC) of mass spectrometry detection of different QC samples. The results showed that the curve overlap of metabolites TIC is high, which means that the retention time and peak intensity were consistent, indicating that the signal stability of mass spectrometry was good when detecting the same sample at different times (**Supplementary Figures 1A, B**). Pearson analysis was performed to evaluate the correlation between biological replicate samples. Pearson’s correlation coefficient (r2) was used as the evaluation index of correlation (**Supplementary Figure 1C**). PCA is a standard unsupervised pattern recognition algorithm, which is mostly used to identify the dominant signals in multi-dimensional data. In this study, PCA was used to examine the overall differences in metabolic profiles among the groups and the variability within groups. The result indicated that the metabolic profiles in each group were clearly clustered and obviously separated, and strong clustering within QC samples (MIX) (**Supplementary Figure 1D**).

### Differential metabolite enrichment analysis

The metabolite data were analyzed with different univariate and multivariate analyses to identify differentially expressed metabolites. Principal component analysis (PCA) and orthogonal partial least squares-discriminant analysis (OPLS-DA) was performed in R (v. 4.1.1) and used to reduce data dimensionality and verify the separation trends between groups. OPLS-DA combines orthogonal signal correction (OSC) and partial least squares-discriminant analysis (PLS-DA) method. In this study, OPLS-DA was performed to decompose the X matrix information (metabolic profile) into Y (groups) correlation and irrelevance by OSC and PLS-DA, thereby screen differential metabolites by removing unrelated differences. The relative contents of the differentially expressed metabolites were normalized and centralized, and K-mean clustering (K-means) analysis was performed to investigate the changing trends of the relative metabolite contents in different samples. The OPLS-DA results yielded variable importance in projection (VIP) for each metabolite, and only those with VIP ≥ 1 were selected. The permutation test was used to evaluate the OPLS-DA model. In addition, metabolites with fold change ≥ 1.5 or ≤ 0.6 were identified as significantly differentially expressed. Their potential functions were investigated using the Kyoto Encyclopedia of Genes and Genomes (KEGG) pathway enrichment analysis. MetaboAnalyst database (https://www.metaboanalyst.ca/) was used to perform the metabolic set enrichment analysis (MSEA).

### Supervised analysis of differential metabolites

The least absolute shrinkage and selection operator (LASSO) regression model and random forest algorithms were used to screen the candidate biomarkers for diagnosing COVID-19 (7, 8). The fivefold cross-validation was applied to assess the power of prognostic classifiers. Briefly, the differential metabolite profiles were first log2 transformed, and the whole cohort was randomly classified into five equal portions. Subsequently, the least absolute shrinkage was used to screen feature selection on four-fifths of the cohort, while the remaining fifth was applied for prediction. In addition, the receiver operating characteristic (ROC) curves were plotted to assess the predictive ability of all five iterations.

### Statistical analysis

Experimental data are presented as mean ± standard deviation, and *P* < 0.05 was considered significant. Continuous parametric variables in the three groups were tested by the analysis of variance, while continuous nonparametric variables were tested using the Kruskal test. Qualitative data analysis was performed using the chi-square test. The above analyses were performed in R version 4.1.1 (R Foundation for Statistical Computing, Vienna, Austria).

## Results

### Identification of differentially expressed metabolites

**Figure 1:**
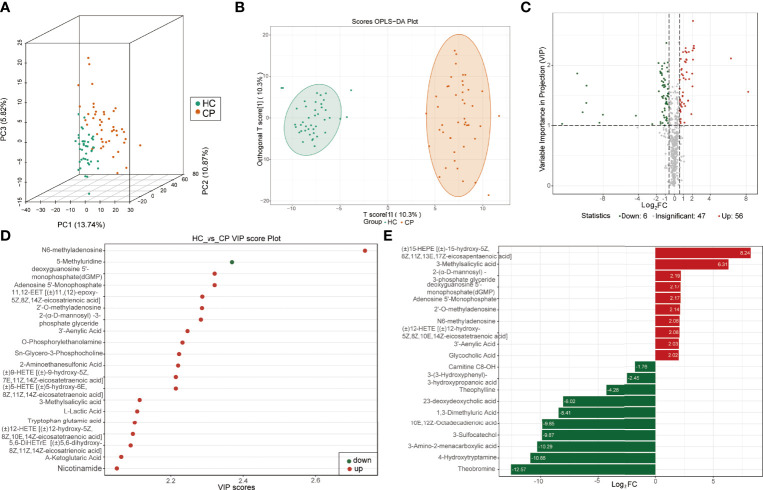
Identification of differentially expressed metabolites between COVID-19 patients and healthy controls. **(A)** Score plots of three-dimension principal component analysis (3D-PCA) discriminating between the metabolic profiles of healthy controls (HC) and COVID-19 patients (CP). **(B)** Orthogonal partial least-squares discriminant analysis (OPLS-DA) score plot of HC and CP. **(C)** Volcanic map of differentially expressed metabolites between HC and CP. Red dots represent upregulated metabolites, gray dots represent unchanged metabolites, and green dots represent downregulated metabolites. **(D)** The top 20 differentially expressed metabolites ranked by the value importance plot (VIP). **(E)** The top 20 differentially expressed metabolites ranked by the log_2_ fold change (Log_2_ FC).

More than 600 metabolites from 128 samples were identified using a widely targeted metabolomics approach based on the UPLC-MS/MS platform and the MetWare database. They belonged to various classes of organic molecules, including amino acids and their metabolites, bile acids, nucleotides and their metabolites, glycerophospholipids, fatty acyls, oxidized lipids, and organic acids and their derivatives. The unsupervised PCA was used to analyze the correlations between healthy controls and COVID-19 patients. The score of three-dimension PCA (3D-PCA) indicated that the metabolites could distinguish between COVID-19 patients and the healthy controls (**Figure 1A**), suggesting high data quality. In addition, the OPLS-DA analysis confirmed a clear tendency towards separating healthy controls from COVID-19 patients (**Figure 1B**). The OPLS-DA model was evaluated using 200 randomized permutations (Q^2 =^ 0.894, *p*<0.005, R^2^Y=0.962, *p*<0.005) and the results showed that OPLS-DA exhibited the optimal discriminatory ability (**Supplementary Figure 2A**). Unlike healthy controls, COVID-19 patients had 125 differentially expressed metabolites (56 upregulated and 69 downregulated) in the plasma (**Figure 1C**). Among the top 20 differentially expressed metabolites between the COVID-19 group and the control group (**Figures 1D, E**), N6-methyladenosine, 3-methylsalicylic acid, 2′-O-methyladenosine, 2-(α-D-mannosyl)-3-phosphate glyceride, dGMP, AMP, and 3′-Adenylic acid were most notable.

**Figure 2:**
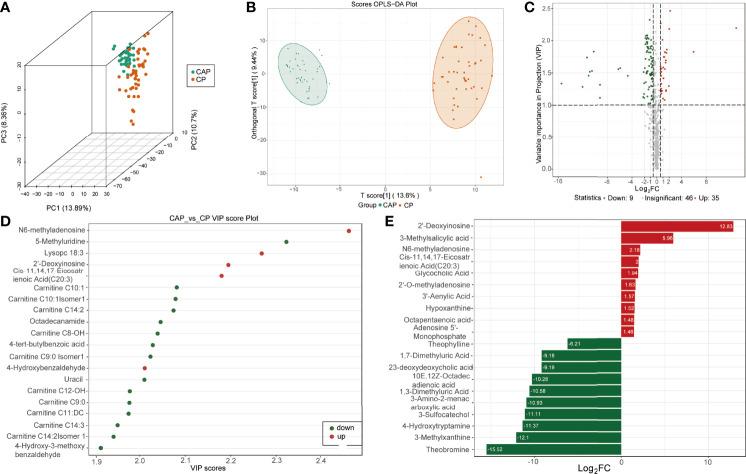
Identification of differentially expressed metabolites between COVID-19 patients and community-acquired pneumonia. **(A)** Score plots of three-dimension principal component analysis (3D-PCA) discriminating between the metabolic profiles of community-acquired pneumonia (CAP) and COVID-19 patients (CP). **(B)** Orthogonal partial least-squares discriminant analysis (OPLS-DA) score plot of CAP and CP. **(C)** Volcanic map of differentially expressed metabolites between CAP and CP. Red dots represent upregulated metabolites, gray dots represent unchanged metabolites, and green dots represent downregulated metabolites. **(D)** The top 20 differentially expressed metabolites ranked by the value importance plot (VIP). **(E)** The top 20 differentially expressed metabolites ranked by the log2 fold change (Log2 FC).

In agreement, the scores of 3D-PCA and OPLS-DA also suggested a clear separation between COVID-19 patients and CAP patients (**Figures 2A, B**). The permutation test (Q^2 =^ 0.945, *p*<0.005; R^2^Y=0.977, *p*<0.005) also indicated that the OPLS-DA model had the best discrimination effect (**Supplementary Figure 2B**). The COVID-19 patients had 134 differentially expressed metabolites (99 downregulated and 35 upregulated) in the plasma (**Figure 2C**). Of these, N6-methyladenosine, 2′-deoxyinosine, and cis-11,14,17-eicosatrienoic acid (C20:3) were most significantly differentially expressed between the two groups (**Figures 2D, E**).

### Metabolic pathway enrichment analysis of differential metabolites

**Figure 3:**
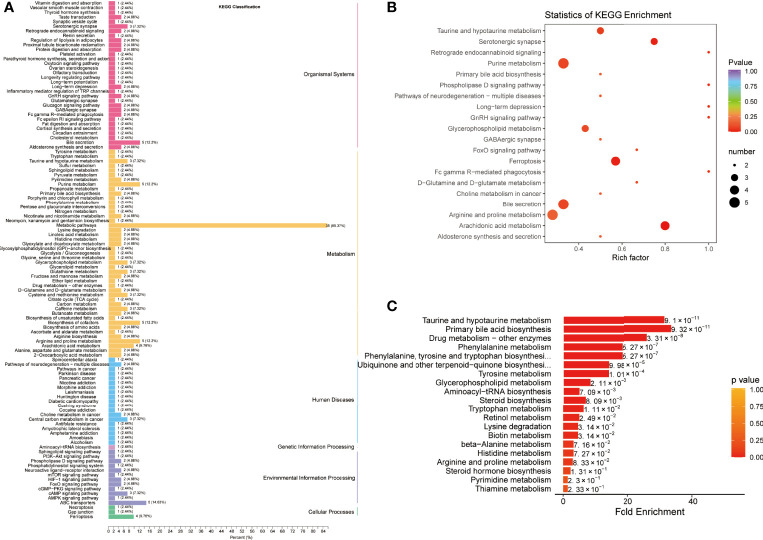
Pathway enrichment analysis of differentially expressed metabolites between COVID-19 patients and healthy controls. **(A)** Kyoto Encyclopedia of Genes and Genomes (KEGG) classification of differentially expressed metabolites between healthy controls (HC) and COVID-19 patients (CP). **(B)** KEGG pathway enrichment analysis of differentially expressed metabolites. **(C)** Metabolite Set Enrichment Analysis (MSEA) between HC and CP.

Pathway enrichment analysis was performed using the KEGG database to elucidate the role of the differentially expressed metabolites in the progression of COVID-19. The KEGG annotation results for the COVID-19 group and the control groups were classified according to the pathway type and divided into 4 categories: organismal systems, metabolism, human diseases, and environmental information processing (**Figure 3A** and **Table S1**). The metabolites expressed only in COVID-19 patients were mainly involved in biliary secretion, ferroptosis, purine, glycerophospholipid, and arachidonic acid metabolism pathways (**Figure 3B** and **Table S2**). To further explore the pathway differences between the COVID-19 group and the control group, MSEA was carried out. It revealed several significantly different pathways such as, taurine and hypotaurine metabolism, primary bile acid biosynthesis, drug metabolism-other enzymes, and phenylalanine metabolism (**Figure 3C** and **Table S3**).

**Figure 4:**
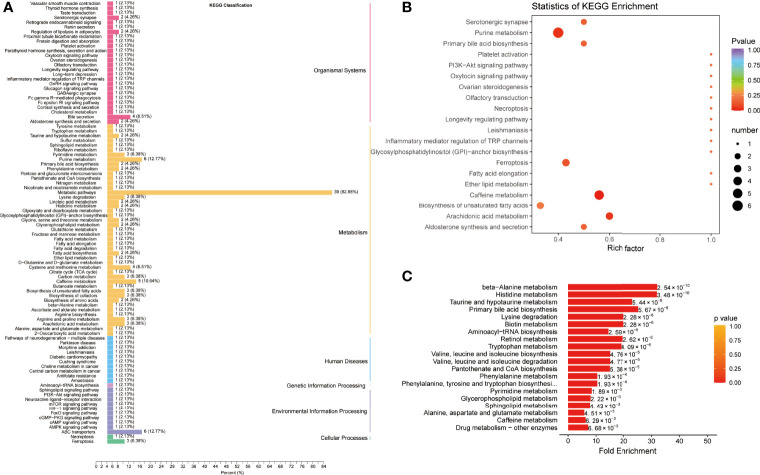
Pathway enrichment analysis of differentially expressed metabolites between COVID-19 patients and community-acquired pneumonia. **(A)** Kyoto Encyclopedia of Genes and Genomes (KEGG) classification of differentially expressed metabolites between community-acquired pneumonia (CAP) and COVID-19 patients (CP). **(B)** KEGG pathway enrichment analysis of differentially expressed metabolites. **(C)** Metabolite Set Enrichment Analysis (MSEA) between CAP and CP.

For the differential metabolites between the COVID-19 group and the CAP group, the KEGG pathways belonged to the same categories identified in the COVID-19 group and the control group (**Figure 4A** and **Table S4**). The enriched pathways were mainly associated with ferroptosis, purine, caffeine, and arachidonic acid metabolism (**Figure 4B** and **Table S5**). Moreover, MSEA uncovered differences in KEGG pathways between the two patient groups: primary bile acid biosynthesis, lysine degradation beta-alanine, histidine, taurine, and hypotaurine metabolism (**Figure 4C** and **Table S6**).

### K-means analysis of differentially expressed metabolites

**Figure 5:**
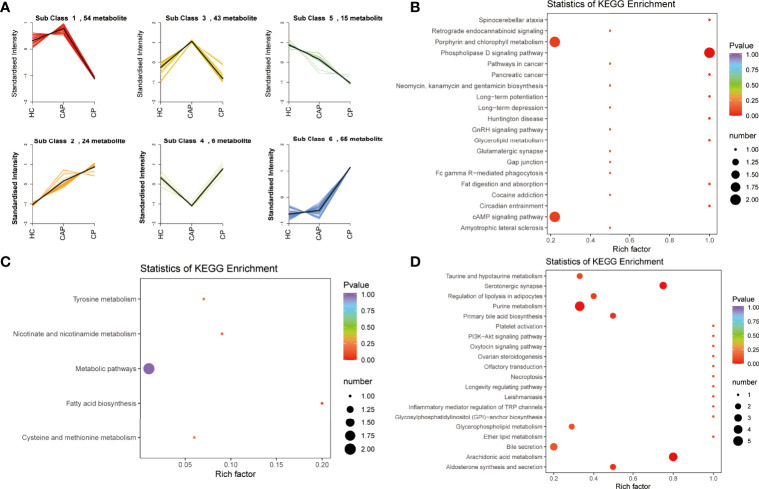
K-mean clustering analysis and pathway enrichment analysis. **(A)** K-means clustering analysis of differentially expressed metabolites across different groups. **(B)** KEGG enrichment analysis of metabolites in subcluster 2. **(C)** KEGG enrichment analysis of metabolites in subcluster 5. **(D)** KEGG enrichment analysis of metabolites in subcluster 6. HC, healthy control; CAP, community-acquired pneumonia; CP, COVID-19 patients.

K-means analysis was performed to determine the variation trend of differential metabolites across the different groups. It revealed significant changing trends divided into six subclasses (**Figure 5A**). Among them, subclass 2, 5, and 6 were in line with expectations, which exhibited increasing or decreasing trends from the HC group to the CAP group to the COVID-19 group. Subsequently, KEGG analysis was performed and showed that the 24 metabolites from subclass 2 were significantly enriched in the phospholipase D and cAMP signaling pathways and porphyrin and chlorophyll metabolism (**Figure 5B** and **Table S7**). The 15 metabolites from subclass 5 were involved in metabolic pathways and fatty acid biosynthesis (**Figure 5C** and **Table S8**). Finally, the 65 metabolites from subclass 6 were associated with arachidonic acid and purine metabolism, taurine and hypotaurine metabolism, serotonergic synapse, and primary bile acid biosynthesis (**Figure 5D** and **Table S9**).

### Biomarker panel for identifying COVID-19 and its model evaluation

**Figure 6:**
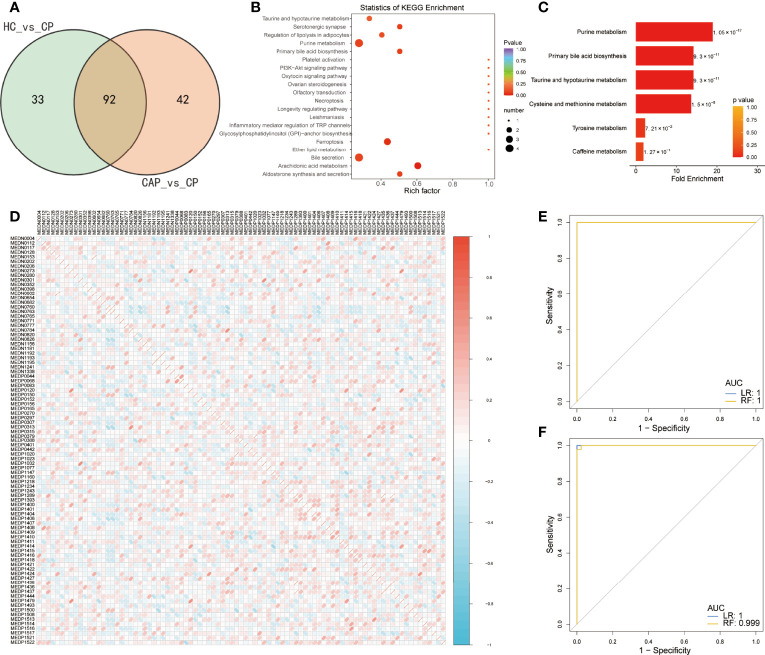
Predictive value of differentially expressed metabolites in predicting COVID-19 patients. **(A)** Venn diagram showing differentially expressed metabolites between two groups: healthy controls (HC) vs. COVID-19 patients (CP) and community-acquired pneumonia (CAP) vs. CP. **(B)** KEGG enrichment analysis of common differentially expressed metabolites. **(C)** Metabolite Set Enrichment Analysis (MSEA) of common differentially expressed metabolites. **(D)** Pearson correlation of 92 differentially metabolites between the two groups. Only the index of metabolites in the database is shown, and detailed metabolic information and classification are listed in **Table S12**. **(E)** Receiver operating characteristic (ROC) curves of the two classifiers based on cross-validation in distinguishing COVID-19 patients (CP) from community-acquired pneumonia (CAP). **(F)** The ROC of the two classifiers based on the cross-validation in distinguishing CP from healthy controls (HC). LR, logistic regression; RF, random forest.

Venn diagrams of mild and severe COVID-19 groups were plotted to characterize the metabolic profile of COVID-19 patients. They showed that the two groups had 92 shared differentially expressed metabolites (**Figure 6A**). These metabolites were enriched with ferroptosis, arachidonic acid metabolism, bile secretion, purine metabolism, and platelet activation KEGG pathways (**Figure 6B** and **Table S10**). Furthermore, MESA indicated that the purine metabolism, primary bile acid biosynthesis, taurine and hypotaurine metabolism, and cysteine and methionine metabolism pathways were significantly differentially expressed between the two groups (**Figure 6C** and **Table S11**). Pearson correlation analysis also revealed a weak correlation between the metabolites (**Figure 6D**). Detailed metabolic information and classification are listed in **Table S12**.

A rigorous machine-learning method was used to explore the predictive value of the differentially expressed metabolites in identifying COVID-19. First, we applied LASSO regression to screen for biomarker metabolites of COVID-19. Seven and fifteen metabolites were identified to discriminate the COVID-19 patients from CAP patients and HC, respectively (**Supplementary Figures 3A, B**). Next, random forest and logistic regression algorithms were used to assess their performance *via* fivefold cross-validation. The ROC analysis showed that the diagnostic model combining seven metabolites performed outstandingly in distinguishing COVID-19 from CAP patients, with an area under the curve (AUC) equaling 1 (**Figure 6E**). In addition, they also performed well in discriminating the COVID-19 group from the control group, with an AUC close to 1 (**Figure 6F**).

### Metabolic profile of severe COVID-19 patients

**Figure 7:**
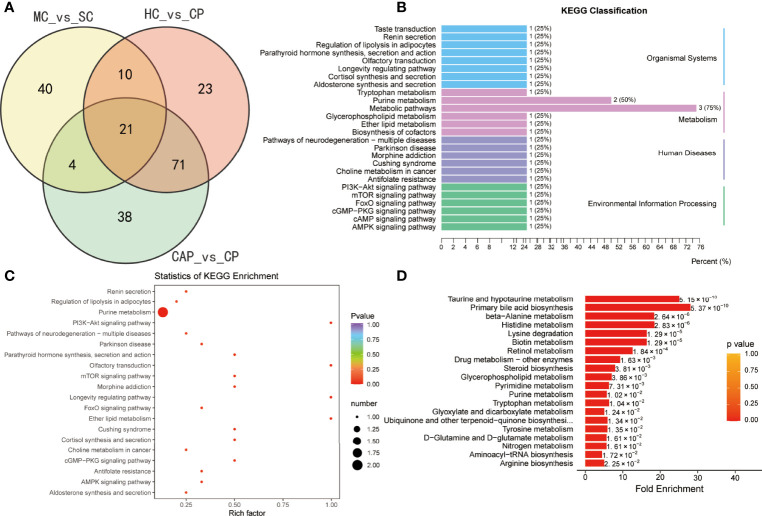
Identification of metabolites specific to severe COVID-19 patients. **(A)** Venn diagram showing differentially expressed metabolites between three groups: healthy controls (HC) vs. COVID-19 patients (CP), community-acquired pneumonia (CAP) vs. CP, and mild COVID-19 (MC) patients vs. severe COVID-19 (SC). **(B)** KEGG classification of common differentially expressed metabolites. KEGG enrichment pathway analysis **(C)** and Metabolite Set Enrichment Analysis (MSEA) **(D)** of 21 common differentially expressed metabolites.

We also performed a differential metabolic analysis of the severe and mild COVID-19 groups to determine the metabolic profile of severe COVID-19. The plotted Venn diagrams of the three groups showed that 21 metabolites were expressed only in severe COVID-19 patients (**Figure 7A**). KEGG pathways were mainly classified into organismal systems, metabolism, human diseases, and environmental information processing (**Figure 7B** and **Table S13**). The enrichment analysis of the differential metabolites revealed they were enriched with purine metabolism, PI3K–Akt signaling, mTOR signaling, and renin secretion pathways (**Figure 7C** and **Table S14**). MSEA also indicated differentially expressed pathways between the groups (**Figure 7D** and **Table S15**): taurine and hypotaurine metabolism, primary bile acid biosynthesis, beta-alanine metabolism, histidine metabolism, and retinol metabolism.

**Figure 8:**
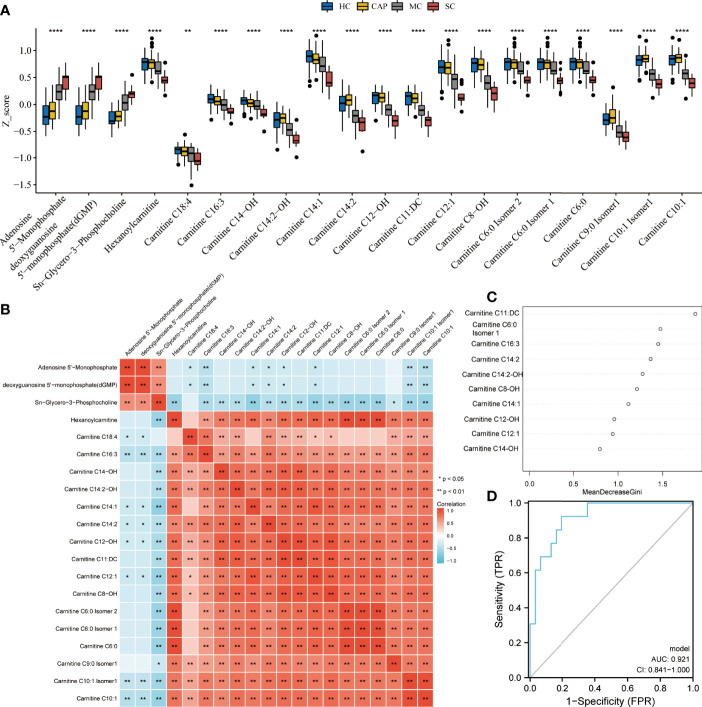
Metabolite performance in predicting severe COVID-19. **(A)** Kruskal test revealing significant differences between all tested groups: healthy controls (HC), community-acquired pneumonia (CAP), mild COVID-19 (MC), and severe COVID-19 (SC) patients. All metabolite profiles were first log2 transformed and then normalized using Z-score standardization. **(B)** Pearson correlation analysis of differential metabolites. **(C)** The top ten ranking metabolites are indicated by random forest. **(D)** Predictive performance of biomarker metabolites evaluated by ROC analysis. *P < 0.05, **P < 0.01, ***P < 0.001, ****P < 0.0001.

Subsequently, the differential analysis showed that AMP, dGMP, and *sn*-glycero-3-phosphocholine were remarkably upregulated in severe COVID-19 patients. By contrast, several carnitine family members were considerably downregulated versus HC subjects, CAP patients, and mild COVID-19 patients (**Figure 8A**). Pearson correlation analysis also uncovered a clear clustering trend among the differentially expressed metabolites. Since a strong correlation existed among the carnitine family members (**Figure 8B**), we applied a random forest algorithm to screen for severe COVID-19 biomarkers (**Figure 8C**). Next, the relative importance of each metabolite was ranked, and the top 10 metabolites (Carnitine C11: DC, Carnitine C6:0 Isomer 1, Carnitine C16:3, Carnitine C14:2, Carnitine C14:2-OH, Carnitine C8-OH, Carnitine C14:1, Carnitine C12-OH, Carnitine C12:1, and Carnitine C14-OH) were selected to construct a diagnostic model for distinguishing between severe COVID-19 patients and mild COVID-19 patients. ROC plots demonstrated that the AUC was 0.921 (0.841–1.000), indicating a relatively high specificity (**Figure 8D**).

## Discussion

In 2020, the COVID-19 pandemic swept the globe with grave effects on human health and the social economy. Since then, governments worldwide have invested in a vast workforce and material resources to improve epidemic prevention measures and prevent the disease (9). Along with the extensive application of vaccines, its spread has been controlled to some extent (10). However, the daily number of new COVID-19 infections remains high, with more than 1 million new infections. Dr. Gregory Poland, head of the Vaccine Research Group at Mayo Clinic, predicted that the disease will continue spreading well into the next century. In addition, efficient, targeted treatments for COVID-19 infection are still lacking (11). Thus, identifying the changes of metabolic molecules and pathways during the viral infection is crucial for improving disease management and accelerating drug development.

Currently, untargeted and targeted metabolomics have been used to reveal the variation of acylcarnitines, fatty acid, triglycerides, and sphingomyelins in COVID-19 patients (12–15). In this study, we also found aberrant expression of amino acid, oxidized lipids, fatty acid, and carnitine in COVID-19 patients. Furthermore, arachidonic acid was identified to be significantly increased in COVID-19 patients, compared to HC. The imbalance of arachidonic acid has been confirmed to be associated with multiple inflammatory pathways (16–18). Multiple studies have indicated that arachidonic acid was related to the infection of the COVID-19 and inflammatory cytokine storm (19, 20). Thus, arachidonic acid deserves further investigation and may be a promising target for COVID-19 therapy. Metabolites exhibited great potential as biomarkers for diagnosing COVID-19. Yamilé López-Hernández et al. identified 3 metabolites combinations that can discriminate between COVID-19 patients and HC, with an AUC of 0.947 (21). Song JW et al. proposed ten metabolites that could distinguish between COVID-19 patients and HC (AUC=0.975) (22). Similarly, Barberis E et al. also revealed the diagnostic value of multiple metabolites with an AUC>0.900 for each model (12). In the present study, seven metabolites were identified based on multiple established machine learning, which can distinguish between COVID-19 patients and CAP patients with an AUC of 1.

In this study, 92 metabolites were specific to COVID-19 patients and included amino acids and their metabolites, fatty acyls, nucleotides and their metabolites, organic acids and their derivatives, and oxidized lipids. Atila, Alptug et al. first described the serum amino acid profile and reported dysregulation of amino acid metabolism in COVID-19 patients (23). Amino acids are precursors of many vital molecules and play a key role in immune cell function (24). Abnormal amino acid metabolism causes neurological symptoms and multiorgan failure. For instance, we know that even mild COVID-19 patients have a certain degree of neurological sequelae after recovering from the infection. Conversely, severe patients with COVID-19 may develop severe multiple organ failure during hospitalization and eventually die. Philips, Paez-Franco, and Rees et al. also studied the role of amino acid metabolism in the progression of COVID-19 infection (25–27). They discovered that the differentially expressed metabolites between the patients and HC were enriched with taurine and hypotaurine metabolic pathways. Taurine has a relatively high abundance in leucocytes and is associated with the inflammatory response (28). Therefore, we speculated that an overactive taurine pathway can drive the excessive immune response in COVID-19 patients. Because amino acids are required for viral replication and virulence, we also proposed amino acid pathways as promising targets for drug development.

The pathway enrichment analysis revealed significantly enriched ferroptosis and energy metabolism pathways in patients with COVID-19. Indeed, the serum of patients with COVID-19 showed an iron imbalance (29). Studies have shown that COVID-19 infection will attack hemoglobin, which is also an important pathogenesis step of COVID-19 (30). This will result in dissociation of the porphyrins from iron and releasing iron from stores into the circulation, causing iron overload and ferritin elevation (31). Bellmann-Weiler R et al. also reported that 88.2% of 259 hospitalized COVID-19 patients had an abnormal iron homeostasis, and 68.8% of anemic patients developed anemia of inflammation (32). Anemia and iron metabolism disorder were significantly associated with increased ICU admission and hospital mortality. Phua, Jason et al. found that serum ferritin levels were significantly elevated in patients with severe COVID-19 compared with mild COVID-19 (33). Furthermore, cellular assays indicated that COVID-19 can decrease the mRNA levels of glutathione peroxidase 4 (34). Most patients with severe COVID-19 disease present with multiorgan damage and failure, and lungs, liver, and kidneys are most affected (35). Ferroptosis is an iron-dependent type of programmed cell death involved in the pathogenesis of various body systems, including the heart, liver, kidney, lung, and intestine (36). Severe organ failure in COVID-19 patients typically starts approximately 14 days post-infection (32). Thus, it coincides with the onset of ferroptosis (37). Metabolomics exhibits robust performance in early diagnosing diseases and monitoring progression. In the present study, we employed multiple well-established machine learning to validate the performance of differential metabolites in diagnosing COVID-19 infection, with an AUC of 1. Therefore, our results suggested that research efforts should focus more on metabolic signatures of the disease.

Twenty-one metabolites were identified only in severe COVID-19 patients. Among them, nucleotides and their metabolites, including AMP and dGMP, and *sn*-glycero-3-phosphocholine were upregulated. In agreement, KEGG analysis demonstrated that purine metabolism pathways were enriched in the severe COVID-19 group. Purine metabolism disorders are responsible for several metabolic diseases. A central component of ATP is AMP, which further hydrolyzes to adenosine. Kondo, Yutaka et al. reported that AMP stimulation can rapidly slow down mitochondrial respiration in mouse and human neurons, thereby reducing dependence on oxygen under a hypo-metabolic state and protecting the brain and other organs from further damage (38). Furthermore, in patients with severe COVID-19, dyspnea and hypoxemia are common symptoms (39). These findings suggested that the AMP increase in severe COVID-19 patients is a self-protection mechanism against hypoxia. It is also worth noting significantly reduced carnitine levels in severe COVID-19 patients. Carnitine is a vitamin-like compound that plays an important role in fatty acid metabolism in granules (40). It is mainly biosynthesized in the kidney, liver, and brain and primarily stored in the skeletal muscle and heart (41). Maintaining carnitine balance is extremely important for regulating and sustaining normal physiological functions, including antioxidant, anti-apoptotic, anti-inflammatory, biomembrane-stabilizing, and anti-fibrosis (40). Low carnitine is associated with multiple diseases, such as advanced liver cirrhosis and cerebral hemorrhage (42). Since patients with severe COVID-19 usually exhibit metabolic disorders and multiple organ dysfunctions, the decrease of serum carnitine in the severe patients may be related to organ dysfunction (43). Furthermore, random forest analysis showed that AMP and carnitine have excellent performance in distinguishing between severe COVID-19 patients and mild COVID-19 patients. These results suggest that understanding the metabolic changes of AMP and carnitine during COVID-19 may advance monitoring disease progression.

However, a few limitations still remain to be noticed in this study. Due to the small number of patients with COVID-19 enrolled in the present study, the diagnostic efficacy of metabolites needs to be further evaluated. Therefore, the results still need further validation in larger prospective cohorts of COVID-19.

Taken together, the present study systematically delineated the serum metabolic profiles in HC subjects, patients with COVID-19, and CAP patients. We demonstrated that the serum metabolites may have a strong potential in identifying COVID-19 patients, especially those with severe disease. This study may provide new insights into COVID-19 diagnosis and treatment.

## Data availability statement

The original contributions presented in the study are included in the article/**Supplementary Material**. Further inquiries can be directed to the corresponding author.

## Ethics statement

The studies involving human participants were reviewed and approved by Yue Bei People’s Hospital. The patients/participants provided their written informed consent to participate in this study.

## Author contributions

JL, Z-BL, Q-QL, YY, S-QZ, P-FK, and FZ collected blood samples and clinical information. JL, Z-BL and J-CL conceived the study and designed the experiment. JL, Z-BL, S-QZ, and J-CL participated in writing the manuscript. All authors read and approved the final manuscript.

## Conflict of interest

The authors declare that the research was conducted in the absence of any commercial or financial relationships that could be construed as a potential conflict of interest.

## Publisher’s note

All claims expressed in this article are solely those of the authors and do not necessarily represent those of their affiliated organizations, or those of the publisher, the editors and the reviewers. Any product that may be evaluated in this article, or claim that may be made by its manufacturer, is not guaranteed or endorsed by the publisher.

## Supplementary material

Supplementary Figure 1 Sample quality control analysis. The overlapped total ion current (TIC) chromatograms of the quality control sample in the positive **(A)** and negative **(B)** modes. **(C)**. Correlation between replicates was assessed using a Pearson correlation test. D. Principal component analysis shows all samples distribution. HC: healthy control, CAP: community-acquired pneumonia, CP: COVID-19 patients, mix represents quality control sample.

Supplementary Figure 2 The validation of OPLS-DA model. **(A)**. The OPLS-DA model for healthy control and COVID-19 patients was validated using permutation test (200 times). **9B)**. The OPLS-DA model for community-acquired pneumonia (CAP) patients and COVID-19 patients was validated using permutation test (200 times).

Supplementary Figure 3 LASSO regression analysis. LASSO regression analysis was applied to screen featured metabolites between the COVID-19, community-acquired pneumonia **(A)** and healthy control groups **(B)**.

## References

[1] Phillips N. The coronavirus is here to stay - here’s what that means. Nature (2021) 590(7846):382–4. doi: 10.1038/d41586-021-00396-2 33594289

[2] Westheim AJF Bitorina AV Theys J Shiri-Sverdlov R. COVID-19 infection, progression, and vaccination: Focus on obesity and related metabolic disturbances. Obes Rev (2021) 22(10):e13313. doi: 10.1111/obr.13313 34269511 PMC8420274

[3] Shen B Yi X Sun Y Bi X Du J Zhang C. Proteomic and metabolomic characterization of COVID-19 patient sera. Cell (2020) 182(1):59–72.e15. doi: 10.1016/j.cell.2020.05.032 32492406 PMC7254001

[4] Thomas T Stefanoni D Reisz JA Nemkov T Bertolone L Francis RO. COVID-19 infection alters kynurenine and fatty acid metabolism, correlating with IL-6 levels and renal status. JCI Insight (2020) 5(14):e140327. doi: 10.1172/jci.insight.140327 PMC7453907 32559180

[5] Rinschen MM Ivanisevic J Giera M Siuzdak G. Identification of bioactive metabolites using activity metabolomics. Nat Rev Mol Cell Biol (2019) 20(6):353–67. doi: 10.1038/s41580-019-0108-4 PMC6613555 30814649

[6] Johnson CH Ivanisevic J Siuzdak G. Metabolomics: beyond biomarkers and towards mechanisms. Nat Rev Mol Cell Biol (2016) 17(7):451–9. doi: 10.1038/nrm.2016.25 PMC5729912 26979502

[7] Tibshirani R. The lasso method for variable selection in the cox model. Stat Med (1997) 16(4):385–95. doi: 10.1002/(SICI)1097-0258(19970228)16:4<385::AID-SIM380>3.0.CO;2-3 9044528

[8] Garge NR Bobashev G Eggleston B. Random forest methodology for model-based recursive partitioning: the mobForest package for r. BMC Bioinf (2013) 14:125. doi: 10.1186/1471-2105-14-125 PMC3626834 23577585

[9] Atzrodt CL Maknojia I McCarthy RDP Oldfield TM Po J Ta KTL. A guide to COVID-19: a global pandemic caused by the novel coronavirus SARS-CoV-2. FEBS J (2020) 287(17):3633–50. doi: 10.1111/febs.15375 PMC7283703 32446285

[10] To KK-W Sridhar S Chiu KH-Y Hung DL-L Li X Hung IF-N. Lessons learned 1 year after SARS-CoV-2 emergence leading to COVID-19 pandemic. Emerg Microbes Infect (2021) 10(1):507–35. doi: 10.1080/22221751.2021.1898291 PMC8006950 33666147

[11] Gavriatopoulou M Ntanasis-Stathopoulos I Korompoki E Fotiou D Migkou M Tzanninis I-G. Emerging treatment strategies for COVID-19 infection. Clin Exp Med (2021) 21(2):167–79. doi: 10.1007/s10238-020-00671-y PMC7598940 33128197

[12] Barberis E Timo S Amede E Vanella VV Puricelli C Cappellano G. Large-Scale plasma analysis revealed new mechanisms and molecules associated with the host response to SARS-CoV-2. Int J Mol Sci (2020) 21(22):8623. doi: 10.3390/ijms21228623 PMC7696386 33207699

[13] Asim M Sathian B Banerjee I Robinson J. A contemporary insight of metabolomics approach for COVID-19: Potential for novel therapeutic and diagnostic targets. Nepal J Epidemiol (2020) 10(4):923–7. doi: 10.3126/nje.v10i4.33964 PMC7812325 33495710

[14] Dogan HO Senol O Bolat S Yildiz SN Buyuktuna SA Sariismailoglu R. Understanding the pathophysiological changes via untargeted metabolomics in COVID-19 patients. J Med Virol (2021) 93(4):2340–9. doi: 10.1002/jmv.26716 33300133

[15] Pang Z Zhou G Chong J Xia J. Comprehensive meta-analysis of COVID-19 global metabolomics datasets. Metabolites (2021) 11(1):44. doi: 10.3390/metabo11010044 33435351 PMC7827862

[16] Kuehl FA Jr. Egan RW. Prostaglandins, arachidonic acid, and inflammation. Science (1980) 210(4473):978–84. doi: 10.1126/science.6254151 6254151

[17] Wang B Wu L Chen J Dong L Chen C Wen Z. Metabolism pathways of arachidonic acids: mechanisms and potential therapeutic targets. Signal Transduct Target Ther (2021) 6(1):94. doi: 10.1038/s41392-020-00443-w 33637672 PMC7910446

[18] Huang N Wang M Peng J Wei H. Role of arachidonic acid-derived eicosanoids in intestinal innate immunity. Crit Rev Food Sci Nutr (2021) 61(14):2399–410. doi: 10.1080/10408398.2020.1777932 32662287

[19] Ripon MAR Bhowmik DR Amin MT Hossain MS. Role of arachidonic cascade in COVID-19 infection: A review. Prostaglandins Other Lipid Mediat (2021) 154:106539. doi: 10.1016/j.prostaglandins.2021.106539 33592322 PMC7882227

[20] Hoxha M. What about COVID-19 and arachidonic acid pathway? Eur J Clin Pharmacol (2020) 76(11):1501–4. doi: 10.1007/s00228-020-02941-w PMC7314570 32583353

[21] Lopez-Hernandez Y Monarrez-Espino J Oostdam AH Delgado JEC Zhang L Zheng J. Targeted metabolomics identifies high performing diagnostic and prognostic biomarkers for COVID-19. Sci Rep (2021) 11(1):14732. doi: 10.1038/s41598-021-94171-y 34282210 PMC8290000

[22] Song JW Lam SM Fan X Cao WJ Wang SY Tian H. Omics-driven systems interrogation of metabolic dysregulation in COVID-19 pathogenesis. Cell Metab (2020) 32(2):188–2020.e5. doi: 10.1016/j.cmet.2020.06.016 32610096 PMC7311890

[23] Atila A Alay H Yaman ME Akman TC Cadirci E Bayrak B. The serum amino acid profile in COVID-19. Amino Acids (2021) 53(10):1569–88. doi: 10.1007/s00726-021-03081-w PMC8487804 34605988

[24] Wu G. Amino acids: metabolism, functions, and nutrition. Amino Acids (2009) 37(1):1–17. doi: 10.1007/s00726-009-0269-0 19301095

[25] Philips AM Khan N. Amino acid sensing pathway: A major check point in the pathogenesis of obesity and COVID-19. Obes Rev (2021) 22(4):e13221. doi: 10.1111/obr.13221 33569904 PMC7995014

[26] Páez-Franco JC Torres-Ruiz J Sosa-Hernández VA Cervantes-Díaz R Romero-Ramírez S Pérez-Fragoso A. Metabolomics analysis reveals a modified amino acid metabolism that correlates with altered oxygen homeostasis in COVID-19 patients. Sci Rep (2021) 11(1):6350. doi: 10.1038/s41598-021-85788-0 33737694 PMC7973513

[27] Rees CA Rostad CA Mantus G Anderson EJ Chahroudi A Jaggi P. Altered amino acid profile in patients with SARS-CoV-2 infection. Proc Natl Acad Sci USA (2021) 118(25):e2101708118. doi: 10.1073/pnas.2101708118 34088793 PMC8237604

[28] Kim C Cha Y-N. Taurine chloramine produced from taurine under inflammation provides anti-inflammatory and cytoprotective effects. Amino Acids (2014) 46(1):89–100. doi: 10.1007/s00726-013-1545-6 23933994

[29] Zhao K Huang J Dai D Feng Y Liu L Nie S. Serum iron level as a potential predictor of coronavirus disease 2019 severity and mortality: A retrospective study. Open Forum Infect Dis (2020) 7(7):ofaa250. doi: 10.1093/ofid/ofaa250 32661499 PMC7337740

[30] Habib HM Ibrahim S Zaim A Ibrahim WH. The role of iron in the pathogenesis of COVID-19 and possible treatment with lactoferrin and other iron chelators. BioMed Pharmacother (2021) 136:111228. doi: 10.1016/j.biopha.2021.111228 33454595 PMC7836924

[31] Zhou F Yu T Du R Fan G Liu Y Liu Z. Clinical course and risk factors for mortality of adult inpatients with COVID-19 in wuhan, China: a retrospective cohort study. Lancet (2020) 395(10229):1054–62. doi: 10.1016/S0140-6736(20)30566-3 PMC7270627 32171076

[32] Bellmann-Weiler R Lanser L Barket R Rangger L Schapfl A Schaber M. Prevalence and predictive value of anemia and dysregulated iron homeostasis in patients with COVID-19 infection. J Clin Med (2020) 9(8):2429. doi: 10.3390/jcm9082429 PMC7464087 32751400

[33] Phua J Weng L Ling L Egi M Lim CM Divatia JV. Asian Critical care clinical trials, g., intensive care management of coronavirus disease 2019 (COVID-19): challenges and recommendations. Lancet Respir Med (2020) 8(5):506–17. doi: 10.1016/S2213-2600(20)30161-2 PMC7198848 32272080

[34] Wang Y Huang J Sun Y Stubbs D He J Li W. SARS-CoV-2 suppresses mRNA expression of selenoproteins associated with ferroptosis, endoplasmic reticulum stress and DNA synthesis. Food Chem Toxicol (2021) 153:112286. doi: 10.1016/j.fct.2021.112286 34023458 PMC8139185

[35] Cheng Y Luo R Wang K Zhang M Wang Z Dong L. Kidney disease is associated with in-hospital death of patients with COVID-19. Kidney Int (2020) 97(5):829–38. doi: 10.1016/j.kint.2020.03.005 PMC7110296 32247631

[36] Stockwell BR Jiang X Gu W. Emerging mechanisms and disease relevance of ferroptosis. Trends Cell Biol (2020) 30(6):478–90. doi: 10.1016/j.tcb.2020.02.009 PMC7230071 32413317

[37] Fratta Pasini AM Stranieri C Girelli D Busti F Cominacini L. Is ferroptosis a key component of the process leading to multiorgan damage in COVID-19? Antioxid (Basel) (2021) 10(11):1677. doi: 10.3390/antiox10111677 PMC8615234 34829548

[38] Kondo Y Sueyoshi K Zhang J Bao Y Li X Fakhari M. Adenosine 5’-monophosphate protects from hypoxia by lowering mitochondrial metabolism and oxygen demand. Shock (2020) 54(2):237–44. doi: 10.1097/SHK.0000000000001440 PMC7044067 31460871

[39] Pascarella G Strumia A Piliego C Bruno F Del Buono R Costa F. COVID-19 diagnosis and management: a comprehensive review. J Intern Med (2020) 288(2):192–206. doi: 10.1111/joim.13091 32348588 PMC7267177

[40] Hanai T Shiraki M Imai K Suetugu A Takai K Shimizu M. Usefulness of carnitine supplementation for the complications of liver cirrhosis. Nutrients (2020) 12(7):1915. doi: 10.3390/nu12071915 PMC7401279 32610446

[41] Vaz FM Wanders RJA. Carnitine biosynthesis in mammals. Biochem J (2002) 361(Pt 3):417–29. doi: 10.1042/bj3610417 PMC1222323 11802770

[42] Flanagan JL Simmons PA Vehige J Willcox MD Garrett Q. Role of carnitine in disease. Nutr Metab (Lond) (2010) 7:30. doi: 10.1186/1743-7075-7-30 20398344 PMC2861661

[43] Wang C Xie J Zhao L Fei X Zhang H Tan Y. Alveolar macrophage dysfunction and cytokine storm in the pathogenesis of two severe COVID-19 patients. EBioMedicine (2020) 57:102833. doi: 10.1016/j.ebiom.2020.102833 32574956 PMC7305897

