# Supplementary figures and images for: Metabolite profile of COVID-19 revealed by UPLC-MS/MS-based widely targeted metabolomics
Source: Front Immunol. 2022 Jul 18;13:894170. doi: 10.3389/fimmu.2022.894170 (PMC9339702; doi:10.3389/fimmu.2022.894170)

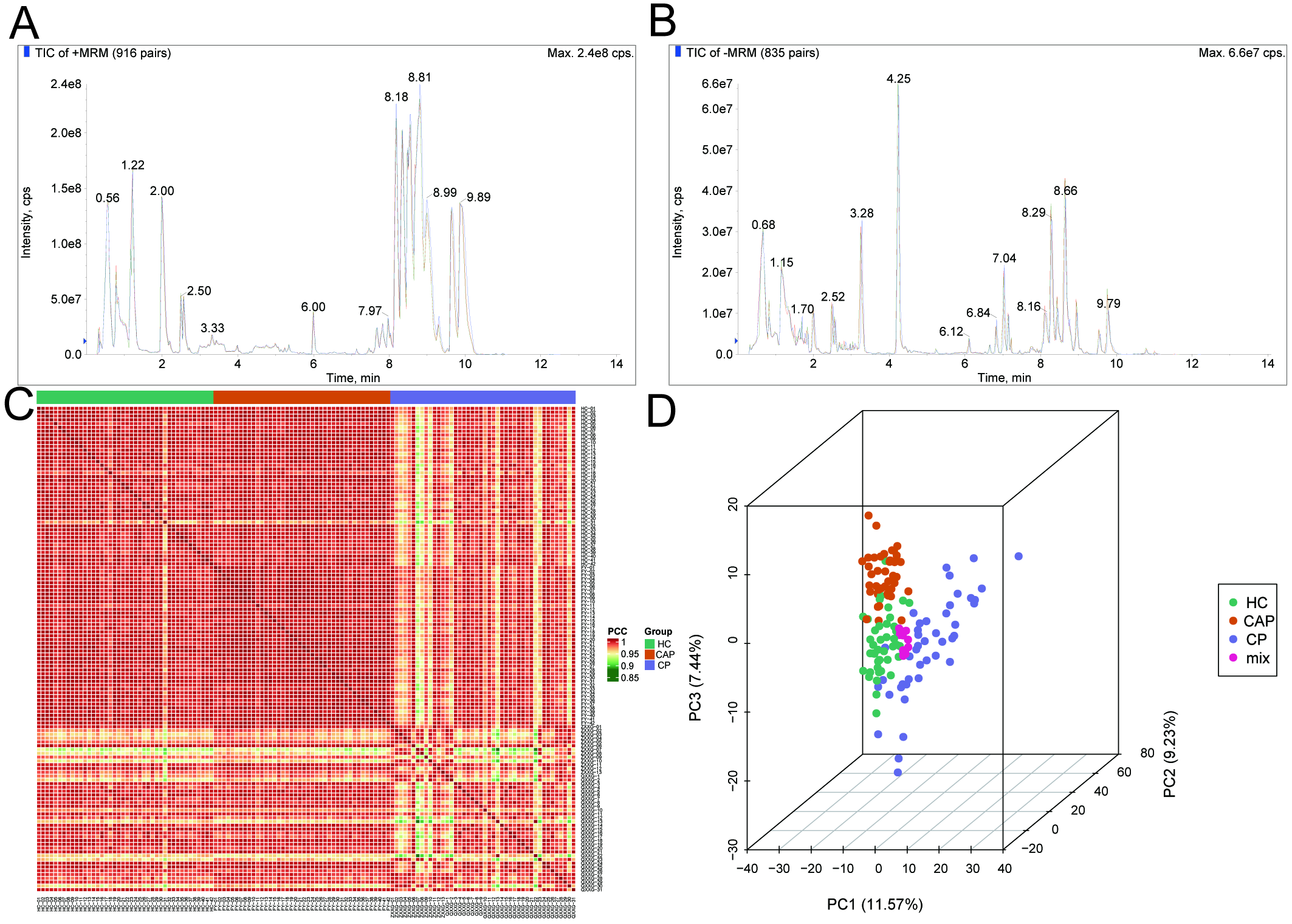

Supplement: Supplementary Figure 1 — Sample quality control analysis. The overlapped total ion current (TIC) chromatograms of the quality control sample in the positive (A) and negative (B) modes. (C). Correlation between replicates was assessed using a Pearson correlation test. D. Principal component analysis shows all samples distribution. HC: healthy control, CAP: community-acquired pneumonia, CP: COVID-19 patients, mix represents quality control sample. [file Image_1.tif]

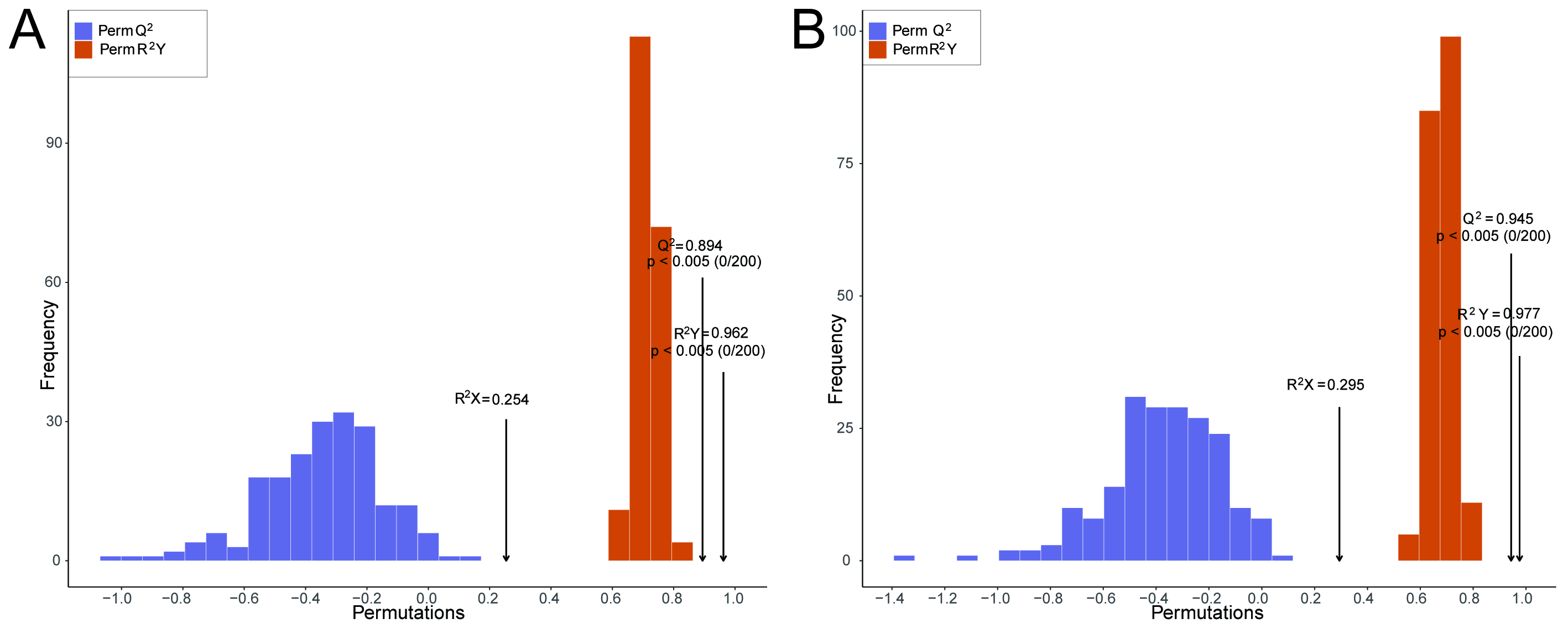

Supplement: Supplementary Figure 2 — The validation of OPLS-DA model. (A). The OPLS-DA model for healthy control and COVID-19 patients was validated using permutation test (200 times). 9B). The OPLS-DA model for community-acquired pneumonia (CAP) patients and COVID-19 patients was validated using permutation test (200 times). [file Image_2.tif]

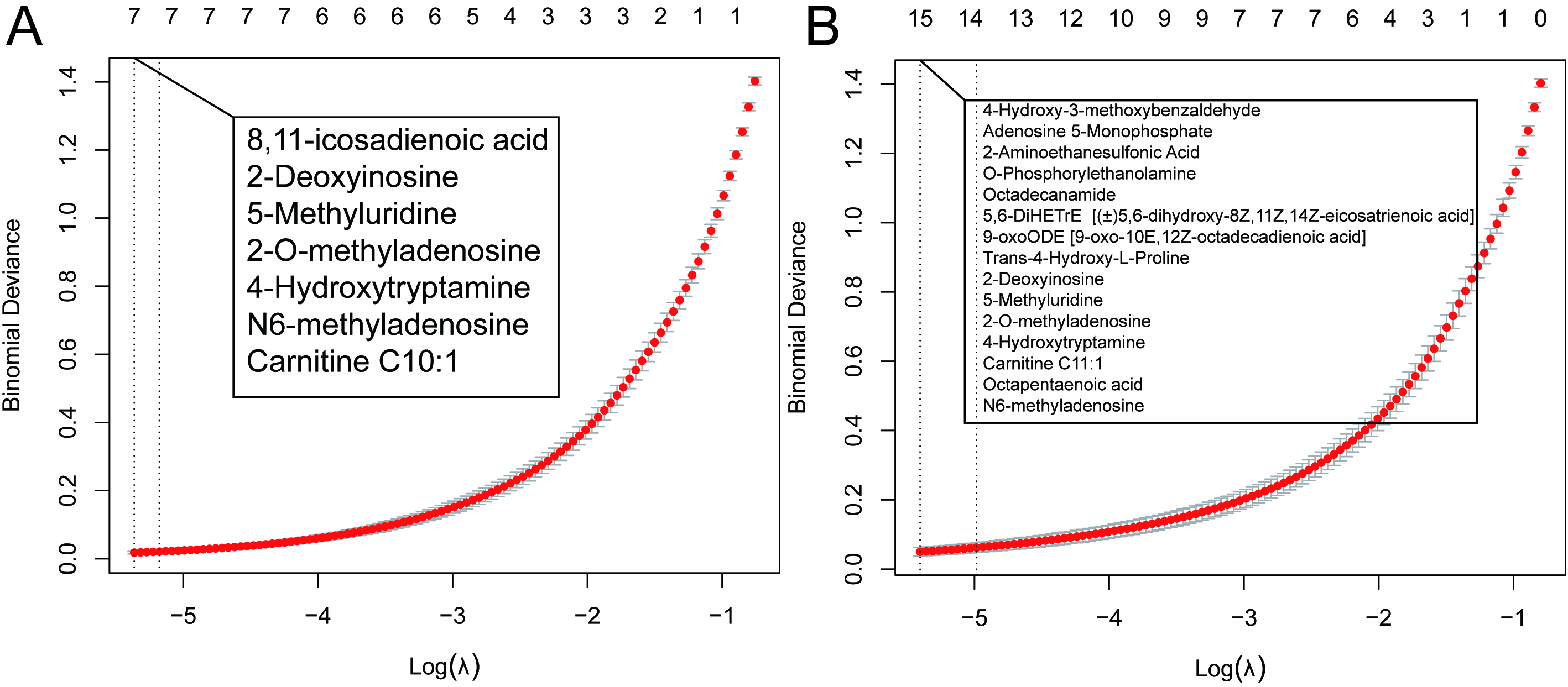

Supplement: Supplementary Figure 3 — LASSO regression analysis. LASSO regression analysis was applied to screen featured metabolites between the COVID-19, community-acquired pneumonia (A) and healthy control groups (B). [file Image_3.tif]
